# Supplementary material for: Exploring Metabolic Adaptations to the Acidic Microenvironment of Osteosarcoma Cells Unveils Sphingosine 1-Phosphate as a Valuable Therapeutic Target
Source: Cancers (Basel). 2021 Jan 15;13(2):311. doi: 10.3390/cancers13020311 (PMC7830496; doi:10.3390/cancers13020311)
Supplement: Supplementary file 1 [file cancers-13-00311-s001.pdf]

Supplementary Materials: Exploring Metabolic Adaptations to the Acidic Microenvironment of Osteosarcoma Cells Unveils Sphingosine 1-Phosphate as a Valuable Therapeutic Target

Margherita Cortini, Andrea Armirotti, Marta Columbaro, Dario Livio Longo, Gemma Di Pompo, Elena Cannas, Alessandra Maresca, Costantino Errani, Alessandra Longhi, Alberto Righi, Valerio Carelli, Nicola Baldini and Sofia Avnet

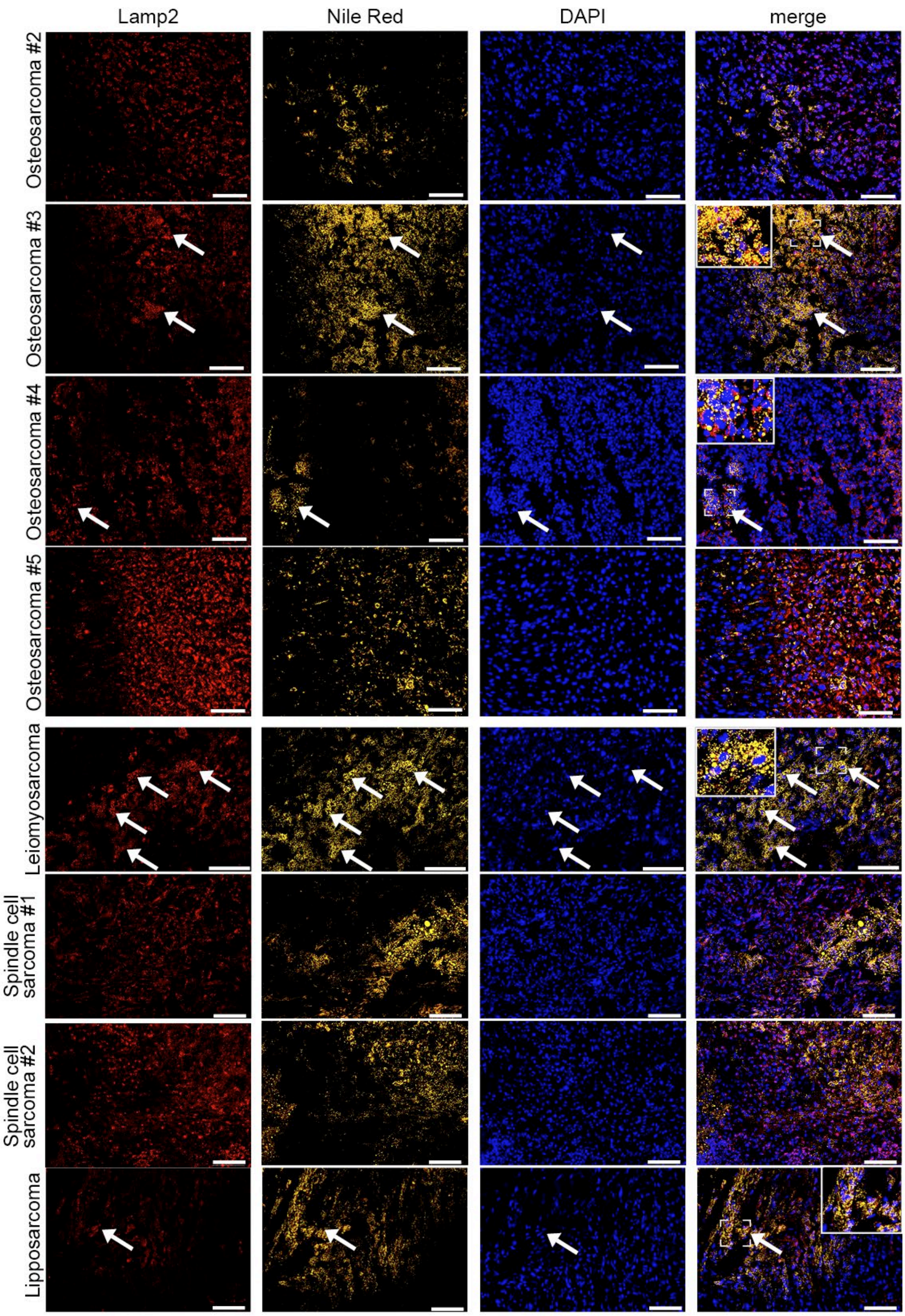

Figure S1. Sarcoma tissues accumulate lipids in acidic regions. Representative IF images of tumor tissues stained with Nile Red (stains LD, yellow), the acidic marker Lamp2 (red), nuclear staining with Hoechst (blue) from patients with OS and with leiomyosarcoma, liposarcoma, and spindle cell sarcoma tissues. Scale bar: 50 μm. Arrowheads indicate cellularized areas of co-localization of Nile Red and Lamp2 staining. Rectangles show magnifications of the highlighted areas.

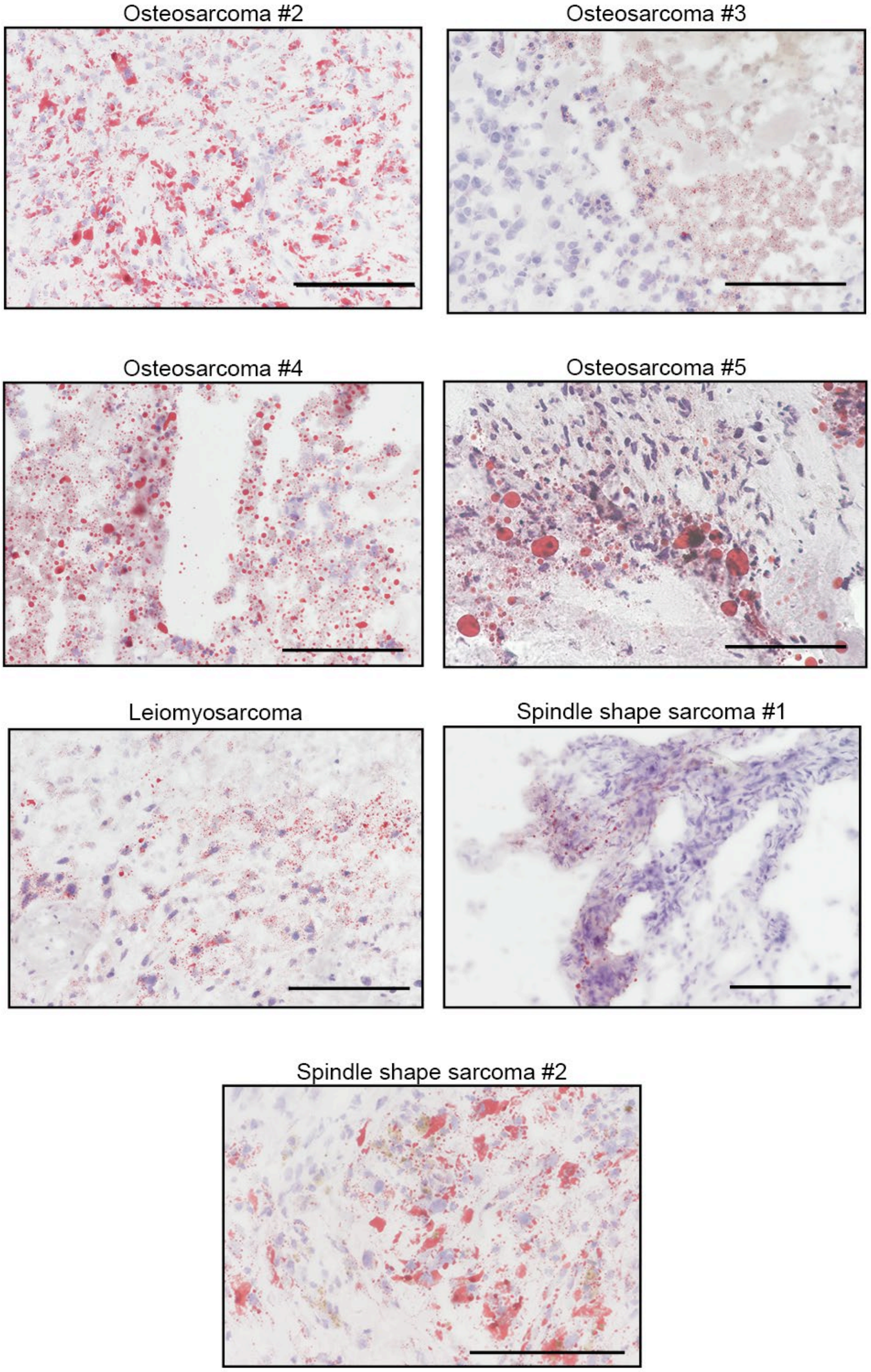

Figure S2. Sarcoma tissues show lipid accumulation. Representative images of Oil Red O staining of tumor tissues (counterstained with hematoxylin). Scale bar 50 µm.

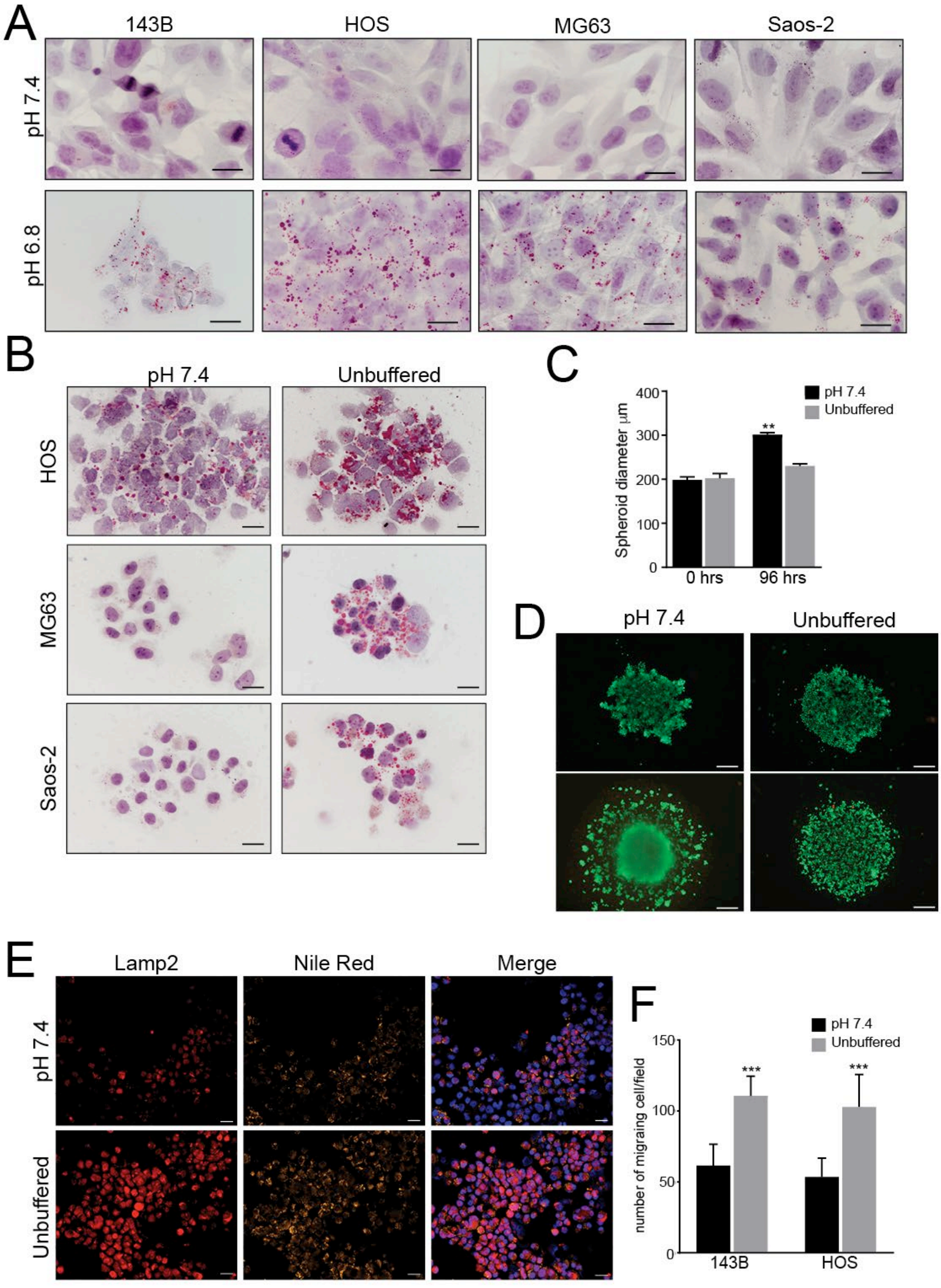

Figure S3. Osteosarcoma cell lines accumulate lipids in 2D and in 3D under acidosis. A-B. Representative images of Oil Red O staining of monolayered cultures treated with acid culture medium (pH 6.8) for 72 h. (**A**) and 3D hanging drop spheroids (**B**) of different OS cell lines (counterstained with hematoxylin). Scale bar 100 µm. (**C**) 143B spheroid diameter at 0 hrs and 96 h. Data are presented as mean ± S.E.M. ** $p < 0.01$ for acid vs neutral. (**D**) Representative live/dead staining of 143B spheroids at 0 and 96 hrs (live cells fluoresce bright green, whereas dead cells with compromised membranes fluoresce red-orange). Scale bar: 50 µm (**E**) Representative IF images of tumor tissues stained with Nile Red (stains LD, yellow), the acidic marker Lamp2 (red), nuclear

staining with Hoechst (blue) from 143B spheroids. Scale bar: 20 µm. (**F**) Quantification of 143B and HOS cell migration (6 h migration of spheroids grown for 5 days) in acid or neutral conditions in Boyden chamber. $n = 6$ samples from 3 independent experiments, unpaired two-tailed Mann Whitney. Data are presented as mean ± S.E.M. *** $p < 0.0001$ for acid vs. neutral.

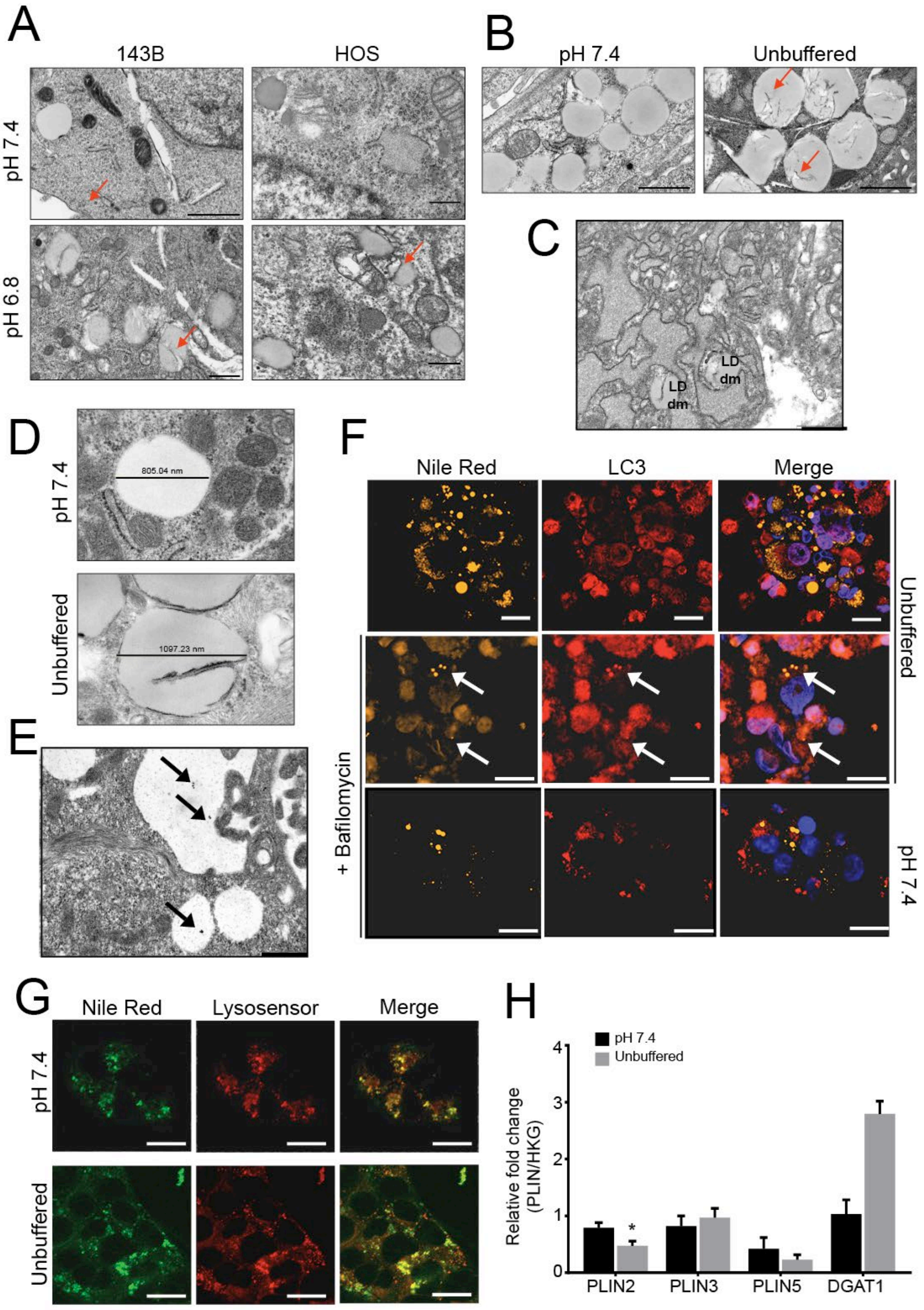

Figure S4. Lipid droplet content is delivered to lysosomes by active lipophagy under acidic conditions. (**A**) EM of cultured 143B and HOS cells in monolayer. Arrowheads show double-membrane-containing LD under acidic conditions. Scale bar 500nm. EM images of cultured HOS spheroids. Arrowheads show double-membrane LD. Scale bar: 2 µm. (**B**) Representative EM images of cultured HOS spheroids showing LD in neutral or acid conditions. Scale bar: 2 µm.(**C**) Tumor tissue

from OS patient visualized by EM. dm: double membrane; scale bar: 1 μm. (**D**) Representative EM images of cultured 143B spheroids showing LD in neutral or acid conditions. Scale bar: 2 μm. (**E**) EM images of immunogold labeling of LC3 on 143B spheroids grown under acidic conditions. Arrowheads indicate gold particles. Scale bar: 500 nm. (**F**) Coimmunostaining of LC3 (red) with Nile Red (orange, stains LD) in cultured 143B spheroids treated with 50 nM bafilomycin A1. Nuclear staining with Hoechst (blue); scale bar: 20 μm; $n = 3$ independent experiments (arrows indicate the co-localized signals). (**G**) Co-localization of Nile Red (green) with LysoSensor (red) in live 143B spheroids. Scale bar: 20 μm; n = 3 independent experiments. (**H**) Real Time PCR analysis of the indicated genes normalized to the quadratic mean of 4 housekeeping genes (HKGs; *GAPDH, GUSB, RNA18S,* and *YWAHZ*). Data presented as mean ± S.E.M. of $n = 6$ RNA samples from 3 independent experiments; unpaired two-tailed Mann-Whitney test; * $p < 0.05$, ** $p < 0.01$ for acid vs neutral.

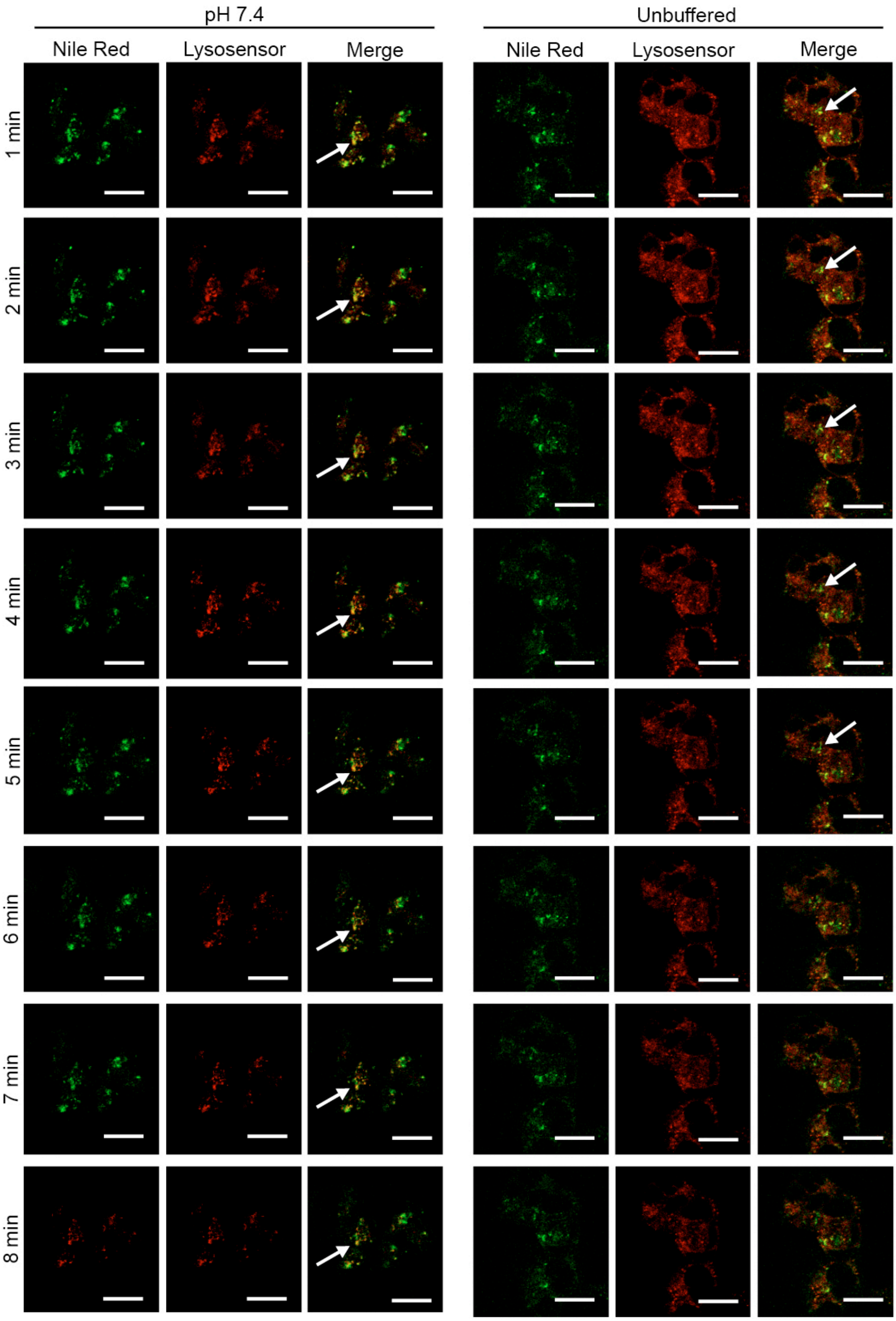

Figure S5. Acidosis reduces the time-length of the interaction between lysosomes and lipid-containing vesicles. Time-lapse magnified images of co-localization of Nile Red (green) with lysosensor (red) in live 143B spheroids. Frames were acquired every 60 sec for 8 min. *n* = 3 independent experiments. Arrowheads indicate areas of co-localization between Nile Red and Lysosensor staining.

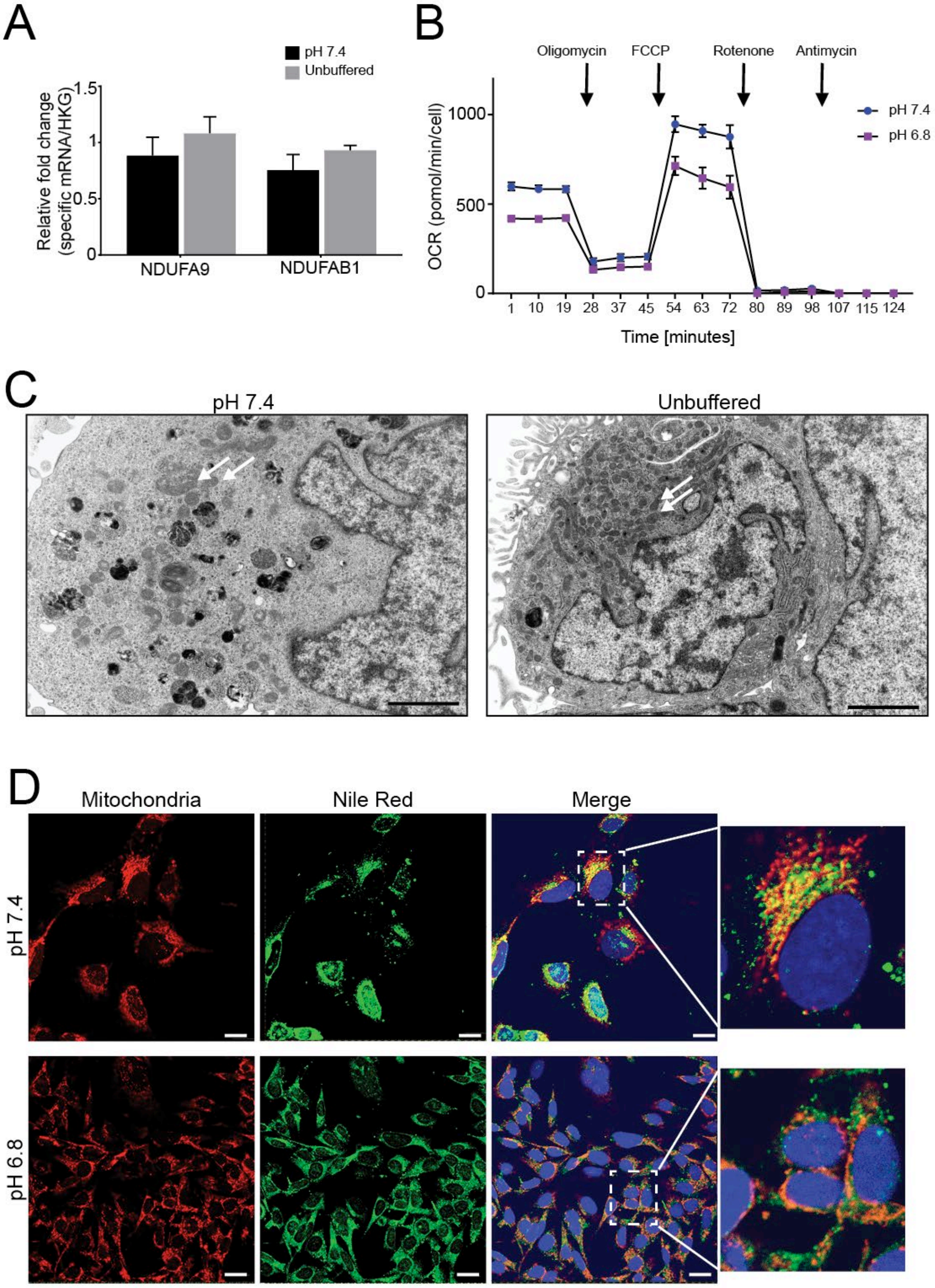

Figure S6. Acidosis increases mitochondrial content that relocalizes to perinuclear regions. (**A**) Real Time PCR analysis of the indicated genes normalized to four housekeeping genes (HKG; GAPDH, GUSB, RNA18S, and YWAHZ) in 143B spheroids cultured for 96 h. Data presented as mean ± S.E.M. of $n = 6$ RNA samples from 3 independent experiments; unpaired two-tailed Mann-Whitney test; * $p < 0.05$, ** $p < 0.01$ for acid vs neutral; **B.** OCR of HOS tumor cells expressed as pmoles O_2/min normalized for protein content, under basal conditions and after injection of oligomycin, carbonyl cyanide 4-(trifluoromethoxy) phenylhydrazone (FCCP), rotenone and antimycin A. Graph shows a representative experiment. (**C**.) EM representative images of cultured 143B spheroids. Arrows indicate mitochondria. Scale bar 2 μm. (**D**.) Co-localization of mitochondria (anti-mitochondrial

surface, red) with LD (Nile Red, green) in HOS cells. Nuclear staining (Hoechst, blue). Right panels show higher magnification of the indicated regions. Scale bar: 20 μm.

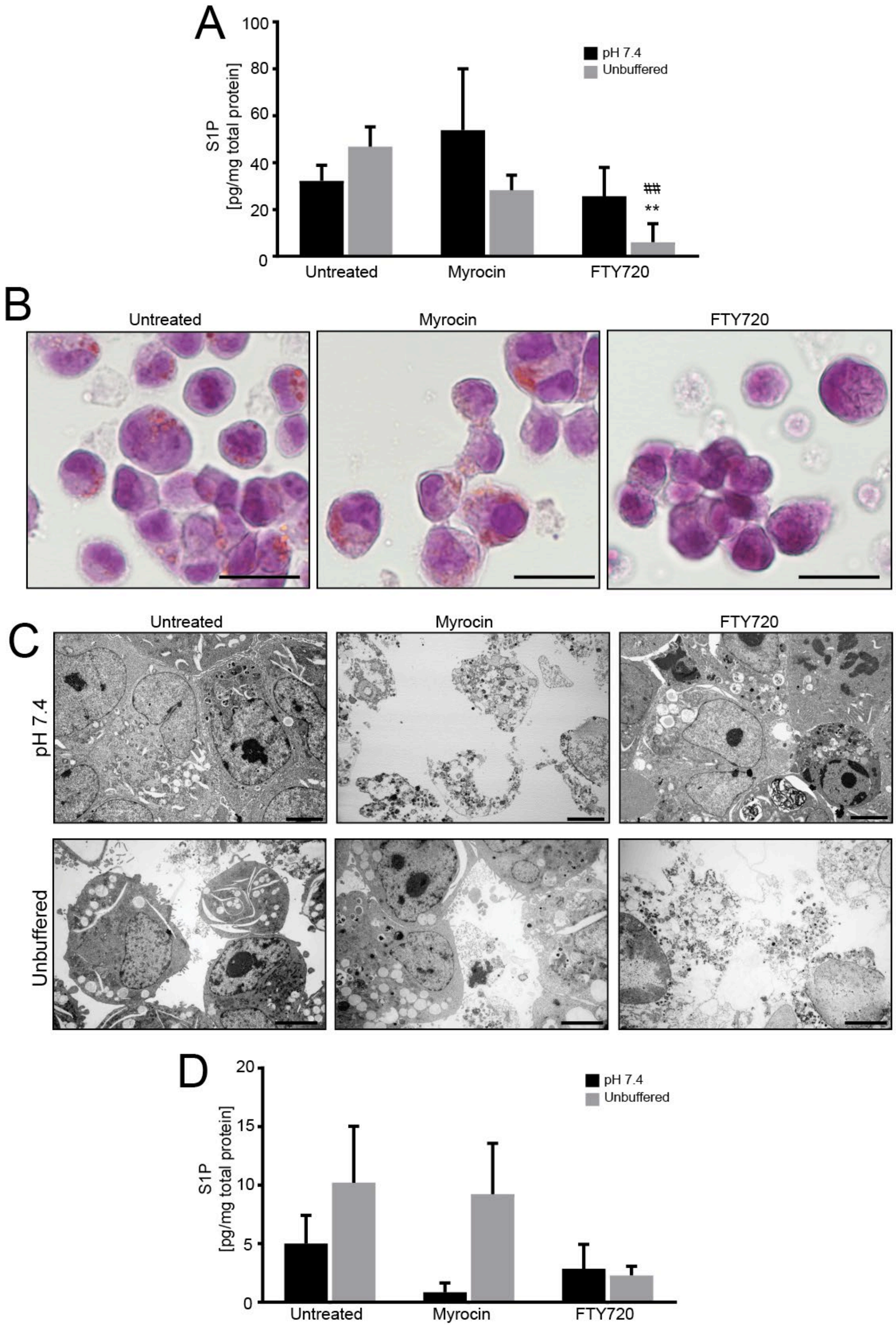

Figure S7. S1P expression and targeting. When indicated, cells were treated with myrocin at 0.75 μM or FTY720 at 0.15 μM. (**A**) S1P expression by ELISA in cultured HOS spheroids treated as indicated. *n* = 6 samples from 3 independent experiments, unpaired *t*-test. Data are presented as mean ± S.E.M. ## $p < 0.01$ vs. untreated at the respective pH condition, ** $p < 0.01$ for acid vs neutral. (**B**) Representative images of Oil Red O staining of cultured 143B hanging-drop spheroids at pH 7.4 treated with myrocin

or FTY720. Scale bar Scale bar 100 µm. (**C**) EM of cultured HOS hanging-drop spheroids treated with myrocin or FTY720. Scale bar: 5 µm. (**D**) S1P expression by ELISA in supernatant of 143B spheroids treated with myrocin or FTY720. $n = 6$ samples from 3 independent experiments, unpaired t-test. Data are presented as mean ± S.E.M.

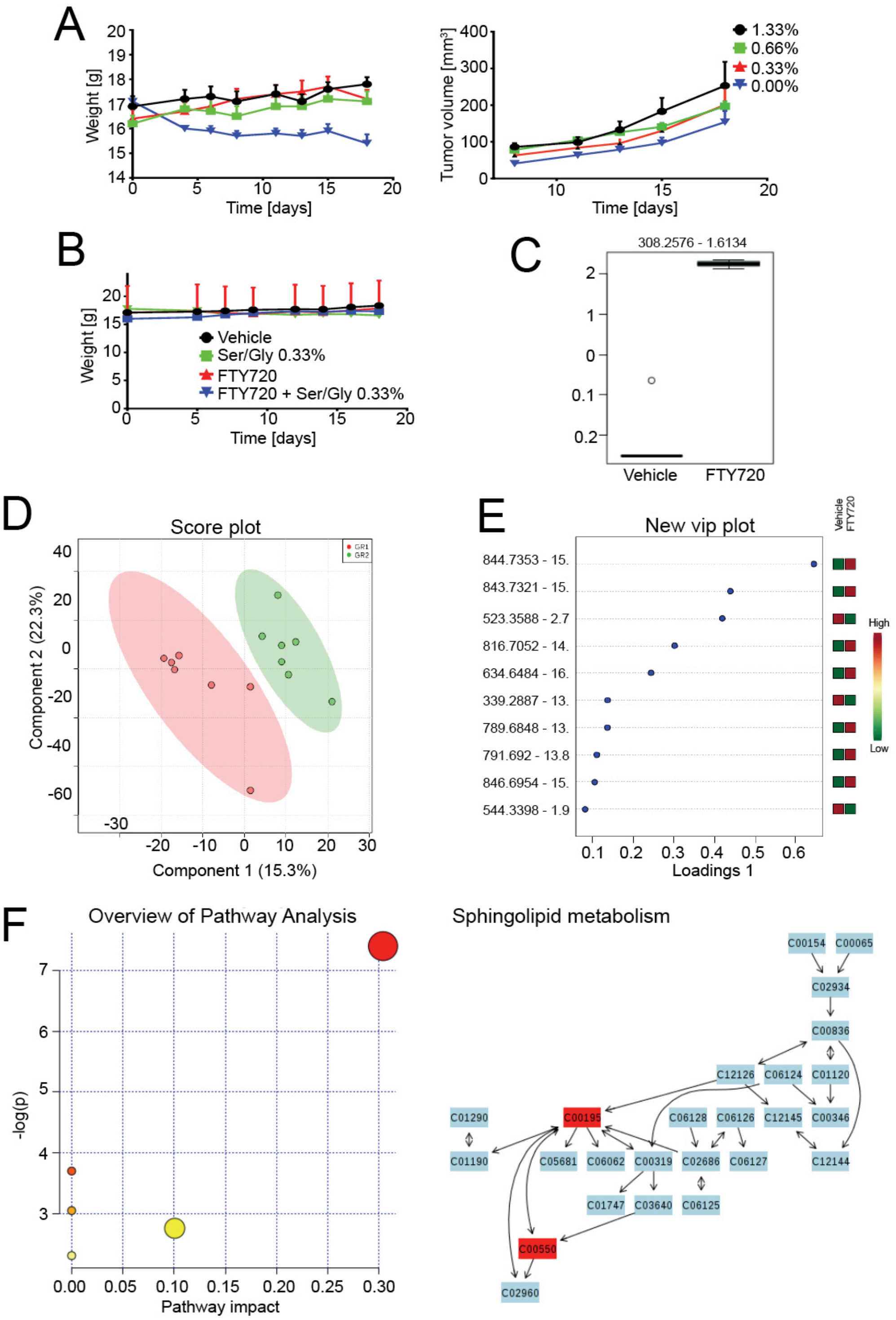

Figure S8. FTY720 treatment targets the tumor and affects its lipid composition without altering mice weight. (**A**) Weight curve and tumor volume of mice treated with different percentage of serine in the diet. (**B**) Weight curve of mice treated with vehicle, Ser/Gly 0.33% diet, FTY720, or the Ser/Gly 0.33% and FTY720 combined. (**C**) Score plot from Partial Least Squares Discriminant Analysis (PLS-DA) of ESI+ lipidomics dataset for mice treated with vehicle (red circle) or FTY720 (green circle); (**D**) Variable Importance in Projection (VIP) plot reporting the 15 most important features that contribute to the separation observed in the PLS-DA analysis. Each feature is labeled as m/z-retention time (min).

There is no real X and Y axes. The plot indicates the ten lipid species (indicated as mass to charge ratio-retention time pairs obtained from the LC-MS dataset) having the biggest impact (top to bottom) on the group separation observed in panel D. The legend on the right indicates whether the given feature is up (red dot) or downregulated (green dot) in the corresponding group. The x axis indicates the relative contribution of each variable to the obtained model. (**E**) Pathway analysis related to the untargeted lipidomics experiment, performed with MetaboAnalyst using the upregulated features (left panel). The plot reveals that sphingolipid metabolism (red dot in the figure) is the only significantly altered pathway (see Table 1 for details). All matched pathways are plotted according to *p*-value from pathway enrichment analysis and pathway impact score from pathway topology analysis. Color gradient and circle size indicate the significance of the pathway ranked by *p*-value (yellow: higher *p*-value and red: lower *p*-value) and pathway impact score (the larger the circle the higher the impact score), respectively. Right panel: schematic representation of the sphingolipid pathway, with the altered individual lipid species represented in red with their HMDB code.

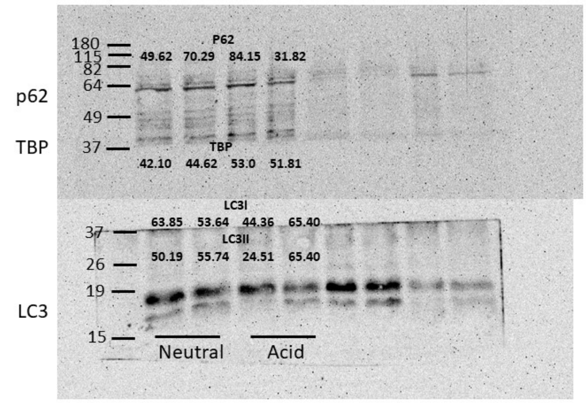

Full unedited gel for Figure 2G

Figure S9. Full unedited gel for Figure 2G.

Table S1. Complete sequences of all the primers.

Gene	Full name	Accession number	Sequence	Probe
GAPDH	Glyceraldehyde 3-phosphate dehydrogenase	NM_002046.3	F:agccacatcgctcagacac R: gcccaatacgaccaaatcc	60
GUSB	β-Glucuronidase	M15182.1|M15182	F: cgccctgcctatctgtattc R: tccccacagggagtgtgtag	57
18S rRNA	18S ribosomal RNA	X03205.1	F: gcaattattccccatgaacg R: gggacttaatcaacgcaagc	48

YWHAZ	Tyrosine 3-monooxygenase/tryptophan 5-monooxygenase activation protein zeta polypeptide	NM_003406.3	F: ccgttacttggctgaggttg R: tgcttgttgtgactgatcgac	9
PLIN1	Perilipin 1	NM_002666.4	F: tgaacattaaagggaagaagttgaa R: ttctcctgctcagggaggt	42
PLIN3	Perilipin 3	NM_005817.4	F: agatgtggctcagctggaa R: gtcccggaacatggtgag	1
PLIN5	Perilipin 5	NM_001013706.2	F: cttgaacacatggacaaagagg R: tcaagttggcctgaataggg	32
DGAT1	Diacylglycerol O-acyltransferase 1	NM_012079.5	F: actaccgtggcatcctgaac R: ataaccgggcattgctca	9
DRP1	Dynamin 1 like	NM_012062.4	F: aaaattgctggtatgaaatgacact R: aaagctccttttgtctcccttt	14
MFF	Mitochondrial fission facto	NM_001277061.1	F: ctcgtagggcataccagcag R: caccacgcaacacaggtct	42
MFN1	Mitofusin 1	NM_033540.2	F: gcctagtggaggttctgttga R: gtgcaaatggaggcaaaact	60
MFN2	Mitofusin 2	NM_014874.3	F: actgcagccaccaagtcc	1

			R: aacctcaattttcttgttcatgg	
NDUFA9	NADH:ubiquinone oxidoreductase subunit A9	NM_005002.4	F: gtcctataccccttggttcctt<br>R: aattcctttggatacatctacgaca	11
NDUFAB1	NADH:ubiquinone oxidoreductase subunit AB1	NM_005003.2	F: cgtgtcctttcagcctatgtc<br>R: cagagagcggtgctgagag	89
SPHK1	Sphingosine kinase 1	NC_000017.11	F: atccagaagcccctgtgtag<br>R: ggtcaggaggtcttcattggt	3
SPHK2	Sphingosine kinase-2	NC_000019.10	F: cactgccctcacctgtctg<br>R: gggattgaccaatagaagcaa	59
BIRC2	Baculoviral IAP repeat containing 2	NC_000011.10	F: gacgtcatcgtgcgtcag<br>R: cgccgacaaggagatacg	70
BIRC3	Baculoviral IAP repeat containing 3	NM_001165.	F: cagcaaggcctgtctgttc<br>R: aatatccccatcccatacagg	79
TRAF2	TNF receptor-associated factor 2	NM_021138.3	F: ggaacacacctgtcccctt<br>R: ggtcgagcagcattaaggtc	9

Publisher's Note: MDPI stays neutral with regard to jurisdictional claims in published maps and institutional affiliations.

Copyright: © 2021 by the authors. Licensee MDPI, Basel, Switzerland. This article is an open access article distributed under the terms and conditions of the Creative Commons Attribution (CC BY) license (http://creativecommons.org/licenses/by/4.0/).
